# Frequency Selectivity in Pulse Responses of Pt/Poly(3-Hexylthiophene-2,5-Diyl)/Polyethylene Oxide + Li^+^/Pt Hetero-Junction

**DOI:** 10.1371/journal.pone.0108316

**Published:** 2014-09-22

**Authors:** Fei Zeng, Siheng Lu, Sizhao Li, Xiaojun Li, Feng Pan

**Affiliations:** Laboratory of Advanced Materials (MOE), School of Materials Science and Engineering, Tsinghua University, Beijing, People’s Republic of China; Institute for Materials Science, Germany

## Abstract

Pt/poly(3-hexylthiophene-2,5-diyl)/polyethylene oxide + Li^+^/Pt hetero junctions were fabricated, and their pulse responses were studied. The direct current characteristics were not symmetric in the sweeping range of ±2 V. Negative differential resistance appeared in the input range of 0 to 2 V because of de-doping (or reduction) in the side with the semiconductor layer. The device responded stably to a train of pulses with a fixed frequency. The inverse current after a pulse was related to the back-migrated ions. Importantly, the weight calculated based on the inverse current strength, was depressed during low-frequency stimulations but was potentiated during high-frequency stimulations when pulses were positive. Therefore, frequency selectivity was first observed in a semiconducting polymer/electrolyte hetero junction. Detailed analysis of the pulse response showed that the input frequency could modulate the timing of ion doping, de-doping, and re-doping at the semiconducting polymer/electrolyte interface, which then resulted in the frequency selectivity. Our study suggests that the simple redox process in semiconducting polymers can be modulated and used in signal handling or the simulation of bio-learning.

## Introduction

Using simple device, simulation of bio-learning has become a popular research topic in the interdisciplinary fields of information, materials and neuroscience. [1]–[3] To further such investigations, researchers must find artificial materials and working models to approximate the bio-learning processes to the greatest possible extent. Dynamic doping in semiconducting polymers has two important features that are very similar to synaptic processes. Firstly, ions, especially alkali metal ions, are a working substance. Secondly, dynamic doping is reversible. Dynamic doping has been applied in both light-emitting electrochemical cells (LECs) and learning or memory cells. [4]–[10] The former is composed of a polymer and electrolyte mixture and undergoing intensive research. In addition, there have been numerous studies regarding the spatial distributions of the ions, charges, and dynamic p-n junctions in dynamic doping. [11]–[15] However, few studies have investigated responses to input frequency or timing during the dynamic doping process, and understanding such responses is imperative for simulating bio-learning processes. Comprehensive investigations are necessary to understand ion migration kinetics at the polymer/electrolyte interface under periodic electric fields. This type of investigation is crucial for evaluating the feasibility of simulating bio-learning on the base of semiconducting polymer/electrolyte systems and dynamic doping processes.

In this paper, we selected Pt/poly(3-hexylthiophene-2,5-diyl) (P3HT)/polyethylene oxide (PEO) + Li^+^/Pt hetero-junction and studied its pulse responses. Currently, P3HT is widely used in polymer field effect transistors and solar cells, [16]–[19] while PEO is widely used as the electrolyte in lithium-ion batteries. [20] Both materials can be easily fabricated in the form of homogenous films using spin-coating and drop-casting. Consequently, a distinct interface can be formed between these materials. [21] Moreover, P3HT can be doped and de-doped when exposed to the ions of a mixed electrolyte and can be easily modified by the doping ions. [22]–[24] A memory device comprised of a P3HT derivative has been formed. [5] Hetero-junction device will be helpful for determining fundamental principles related to ionic migration kinetics under small-amplitude periodic pulse stimulations (the preferred type of stimulation used in neuroscience).

During our investigation, we identified an interesting phenomenon: the pulse response was frequency dependent. Electric measurements showed that the direct current (DC) characteristics were not symmetric between –2 and +2 V. In particular, negative differential resistance (NDR) existed in the input range of 0 to 2 V but not between –2 to 0 V; this asymmetry resulted from de-doping (or reduction) in the semiconductor layer. The pulse stimulation indicated that a single pulse response contained two parts: one part was the current during the pulse width, and the other was the discharging current after the pulse. The latter was dominated by inversely migrating ions. The responses were usually stable after several inputs, such that a fixed frequency determined the responses. Using both the triangular and rectangle pulse stimulations with amplitudes of about 0.3 to 0.5 V, we calculated the weight variation according to the discharging amplitude as a function of the input frequency. The weight was usually depressed at lower frequencies (<50 Hz) but was potentiated at higher frequencies (>50 Hz) when the positive pulses were loaded; this finding demonstrated frequency selectivity. The mechanism governing the observed phenomena was analyzed and a model was proposed in which the input frequency modulated the dynamic doping at the polymer/electrolyte interface. We presumed that such frequency selectivity could be used in signal handling or even for performing synaptic computation in bio-learning [25].

## Materials and Methods

### 1. Materials

**Figure 1:**
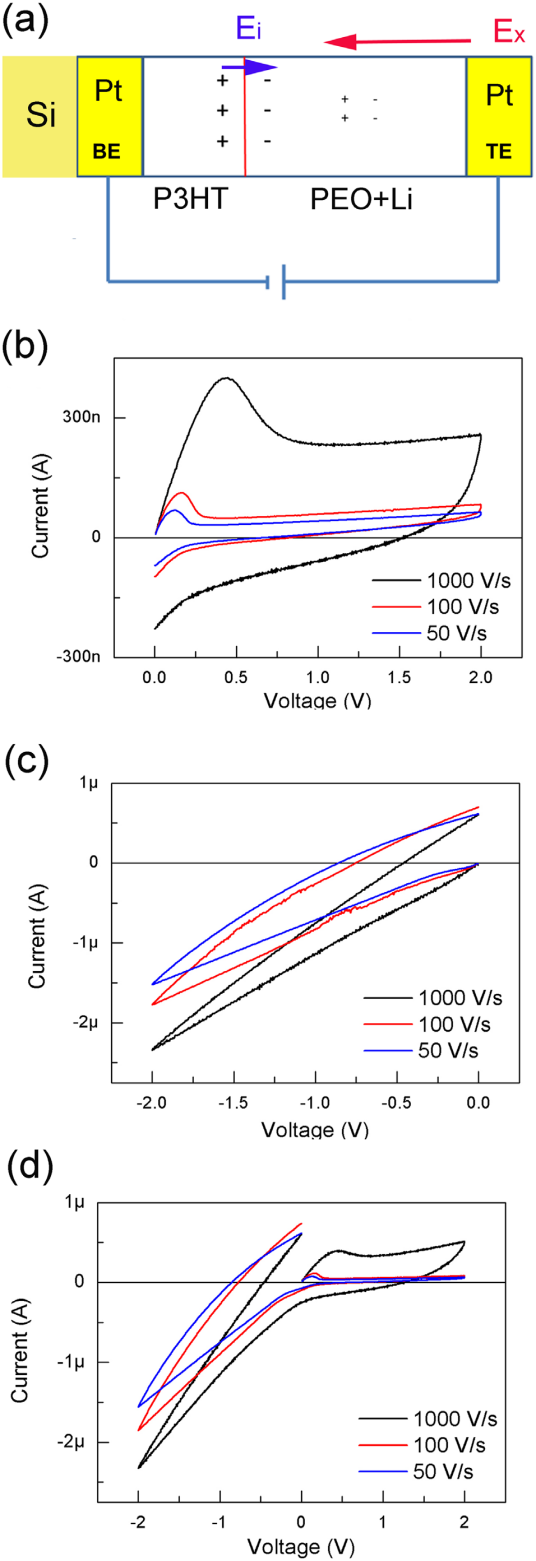
Materials, device structure and DC properties. (a) Schematic diagram of device structure, ion migration and electric field distribution under bias. The label BE and TE refer to bottom electrode and top electrode, respectively. The larger ‘+’ and ‘–’ refer to Li^+^ and CF_3_SO_3_^−^, respectively, at the interface. E_i_ is the internal electric field composed of Li^+^ and CF_3_SO_3_^−^ pairs at the interface and E_x_ is the external electric field. I–V curves were obtained by DC sweeping. The sweeping direction were (b) 0 → 2 → 0 V, (c) 0 →–2 → 0 V, and (d) 0 → 2 →–2 → 0 V, respectively. The sweeping rates are labelled in the figure.

P3HT was purchased from Zhejiang Optical & Electronic Technology Co. Ltd. PEO (MW = 100000) and lithium trifluoromethanesulfonate (LiCF_3_SO_3_) were purchased from Sigma-Aldrich Co. Ltd. These substances were used as received. The Pt/P3HT/PEO + Li^+^/Pt device was fabricated using following methodology. First, a 100 nm Pt film was deposited on a Si substrate and was used as the bottom electrode (BE). Second, a 3-µL dichlorobenzene solution of P3HT was spin-coated on the BE and heated inside a glove box filled with N_2_. The dichlorobenzene solution of P3HT was spin-coated onto the BE at 500, 3000, and 1500 rpm for 10, 30, and 20 s, respectively. The P3HT film was baked at 100°C for 1 h, and then at 140°C for 20 min; its thickness was about 25 nm. Third, a 3-µL aqueous solution of the PEO and LiCF_3_SO_3_ was drop cast on the P3HT film and baked at 60°C for 20 min. Finally, the Pt top electrodes (TEs) with a thickness of 80 nm and a diameter of 300 µm were deposited on the PEO + Li^+^ layer with electron beam deposition using a shadow mask. A schematic of device structure is shown in Figure 1a.

### 2. Measurements

The electrical characteristics of the devices were determined using a semiconductor device analyzer (B1500A, Agilent) and an arbitrary function generator (B1530, Agilent). For all measurements, the BE was grounded. Raman spectra of the polymer films were obtained using an HR-800 Raman system. With a resolution of 1 cm^−1^, a 532 nm He-Ne laser was used as the excitation source.

## Results and Discussions

The device’s DC properties were examined and are shown in Figures 1b to 1d. All DC curves show hysteresis loops, regardless of bias sign. However, the shapes of the hysteresis loops varied depending on the sweeping rate. As the sweeping rate increased, the loop area increased. The residual current values indicated that the ion current was slower than the sweeping rate. In addition, faster sweeping rates produced larger residual currents. The device may be regarded as an ion memcapacitive system because its behaviors apparently depend on processing history and ion migration. [26] This device differs from a typical TiO_x_ memristor because, after the sweeping process, the original electrolyte balance was restored, and the state during sweeping was forgotten [27].

NDR could be observed when the bias was positive (Figure 1b). The bias that resulted in NDR (V_NDR_) increased as the sweeping rate increased. Ranging from 0.2 to 0.5 V when the sweeping rate was 100 V/s, V_NDR_ varied for each individual device, relative to the real ion concentrations in the individual cell. The NDR effect was not observed during negative DC sweeping (Figure 1c). If the bias was swept using a cycle of 0 → 2 → 0 →–2 → 0 (Figure 1d), the NDR effect was weakened after the first sweeping cycle. However, the NDR effect in Figure 1b could be recovered if only the positive bias was loaded, regardless of any previous operation. The results in Figure 1b to 1d imply a selective ability to input signals.

**Figure 2:**
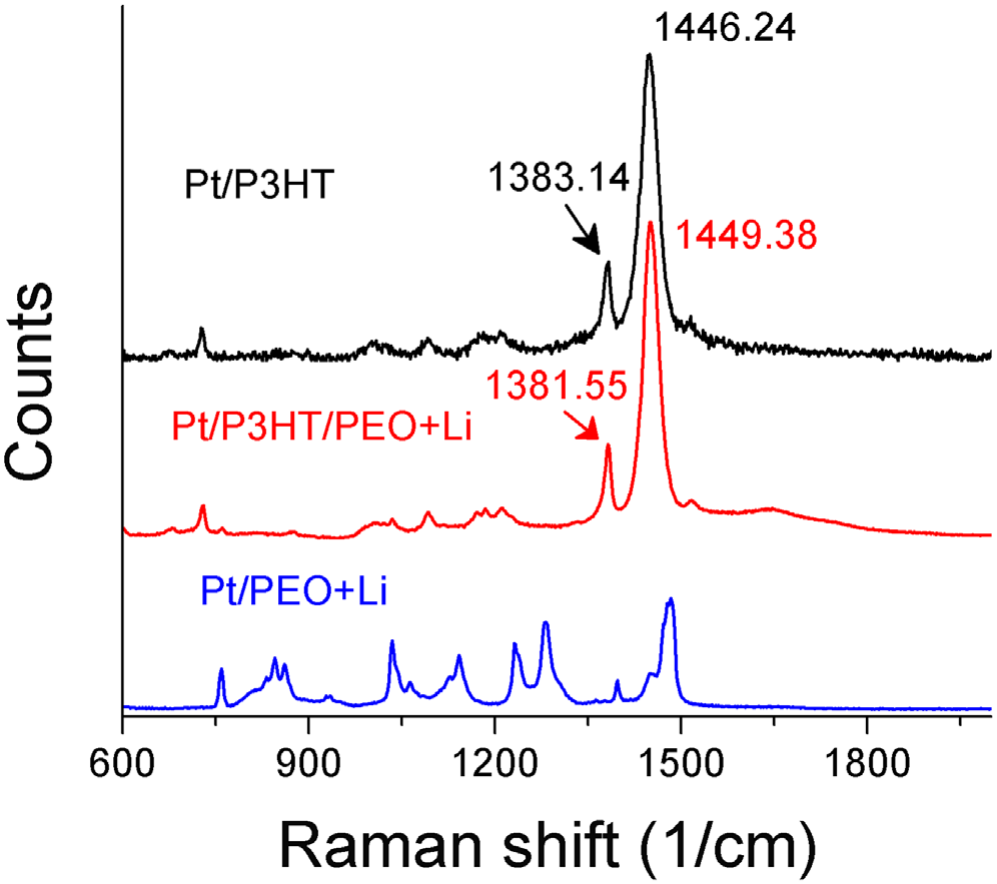
Raman spectra for Pt/P3HT, Pt/PEO + Li^+^, and Pt/P3HT/PEO + Li^+^.

The NDR effect should relate to the doping and de-doping of P3HT. In situ Raman observations have indicated that polarons would be generated by the injected carriers. [22] If P3HT were oxidized by FeCl_3_, a polaron peak at 1405 cm^−1^ would appear. If the P3HT layer were not completely oxidized, the characteristic peak of 1446 cm^−1^ would be red shifted to about 1414 cm^−1^. When the P3HT derivative was composed of a double layer with electrolyte PEO, the initial state might be slightly doped by the anions in the PEO layer. [5] In the present study, we examined the initial state of the P3HT single layer, the P3HT/PEO + Li^+^ double layer, and the PEO + Li^+^ single layer. The Raman spectrum of the single P3HT layer was consistent with that in Ref. 22 as shown in the upper part of Figure 2. When the P3HT/PEO + Li^+^ double layer formed, the signals from PEO + Li^+^ overlapped the signals from P3HT (the middle part of Figure 2), which produced a slight shift in the characteristic peak from 1446 to 1449 cm^−1^ and from 1383 to 1381 cm^−1^. The ratio of I (1446 cm^−1^)/I (1383 cm^−1^) was about 3.03, while the ratio of I (1449 cm^−1^)/I (1381 cm^−1^) was 3.35. This ratio did not significantly change after the PEO + Li^+^ layer overlapped, which indicated that the P3HT layer would be mainly neutral when polarons did not form in our device’s initial state.

Early study of LECs containing LiCF_3_SO_3_ salt indicated that the applied voltage could drive ions to migrate to the electrodes and, subsequently, form thin electric double layers at the interfaces. [28] This finding was seemly consistent with our observation of NDR resulting from a reversed electric field. However, the LECs were actually bulk hetero-junctions. Study of gate-controlled LECs using LiCF_3_SO_3_ salt indicated that the n-doping resulted in high mobility and a high channel current, and the light-emitting zone approached the cathode electrode. P-doping resulted in smaller mobility and a lower channel current, and the light zone crossed over a wider lateral length. [29] This finding suggested a difference in motility between cations and anions of CF_3_SO_3_^−^ in the semiconducting polymer. Molecular dynamics simulations were used to investigate the migration of ions through poly(3-octylthiophene) and indicated that the positive charges on the polymer’s thiophene rings and the negative charges on the anions served to bind the host and dopant subsystems to form domains of short-term order, in which the ions were intercalated between the planar conjugated backbones of the polymer. Application of electric fields along the channel directions also induced anion motion, but applying fields perpendicular to the channel destroyed the lattice order. [30] In our samples, the P3HT layer did not grow in an ordered direction, and consequently, the migration of anions was be easily hindered. Therefore, based on the Raman analysis, DC I–V curves and experiments conducted by other groups, we concluded that the NDR phenomenon originated from the processes described below.

When a small positive DC bias was loaded on the TE, the Li^+^ migrated toward the BE, while the CF_3_SO_3_^−^ should migrate in the opposite direction. The mobility of Li^+^ is higher and these ions are attractive to CF_3_SO_3_^−^. In this case, the ionic states in the PEO + Li^+^ layer resembled plasma. The Li^+^ entered into the P3HT layer at the P3HT/PEO + Li^+^ interface, which resulted in the doping of the P3HT layer. However, the migration of CF_3_SO_3_^−^ was restricted at the polymer/electrolyte interface because of the larger molecular size and mass. Therefore, the Li^+^ accumulated at the polymer side, while the CF_3_SO_3_^−^ accumulated at the side with the electrolyte layer. This process established an internal electric field, E_i_, that was opposite to external field E_x_ (Figure 1a). When E_i_ was larger than E_x_ because of ions accumulated at the interface, discharging occurred at the interface, which led to the NDR phenomenon in Figure 1b. This discharging process included the effects of parasitic capacitance and ion migration driven by the inverse electric field. At this time, de-doping occurred at the side with the P3HT layer. If the bias were negative, the loaded bias value (<2 V) was not sufficient to effectively dope the CF_3_SO_3_^−^ into P3HT. Therefore, the NDR effect could not appear for a negative bias.

Internal field E_i_ could be simply estimated using the NDR effect. The total thickness of the polymer and electrolyte layers was about 1 µm, and consequently, the internal field was about 3000 to 5000 V/cm. However, the ions usually migrated along the backbone channel in the electrolyte. [31] Therefore, the effective internal field at the polymer/electrolyte interface should be quite higher than the above estimated value. If the areas that the backbone direction is parallel to the normal of the P3HT layer were about 1/10 of the electrode area, the internal field would be enhanced by an order of magnitude. We sought to find a method to characterize the effective areas in which the ions were directed along the backbone channel of the electrolyte into the P3HT layer.

Apart from doping and de-doping in semiconducting polymer, [5], [6], [32] many factors may influence the NDR phenomenon, including ions concentrations, the type and temperature of substrates, additives (or impurities), and test temperature. [33] Impurities introduced into the experimental process would mostly influence our experimental results. Usually, ion migration predominates in PEO electrolytes. [34] Our device contains semiconducting polymer and PEO electrolyte double layers, and the latter determines the device’s migration kinetics. A block copolymer is composed of networks of P3HT and PEO passing through each other, such that, the polymer’s conductivity is simultaneously electronic and ionic. [35] Consequently, the processes we observed were principally dominated by ion migration with lower mobility.

**Figure 3:**
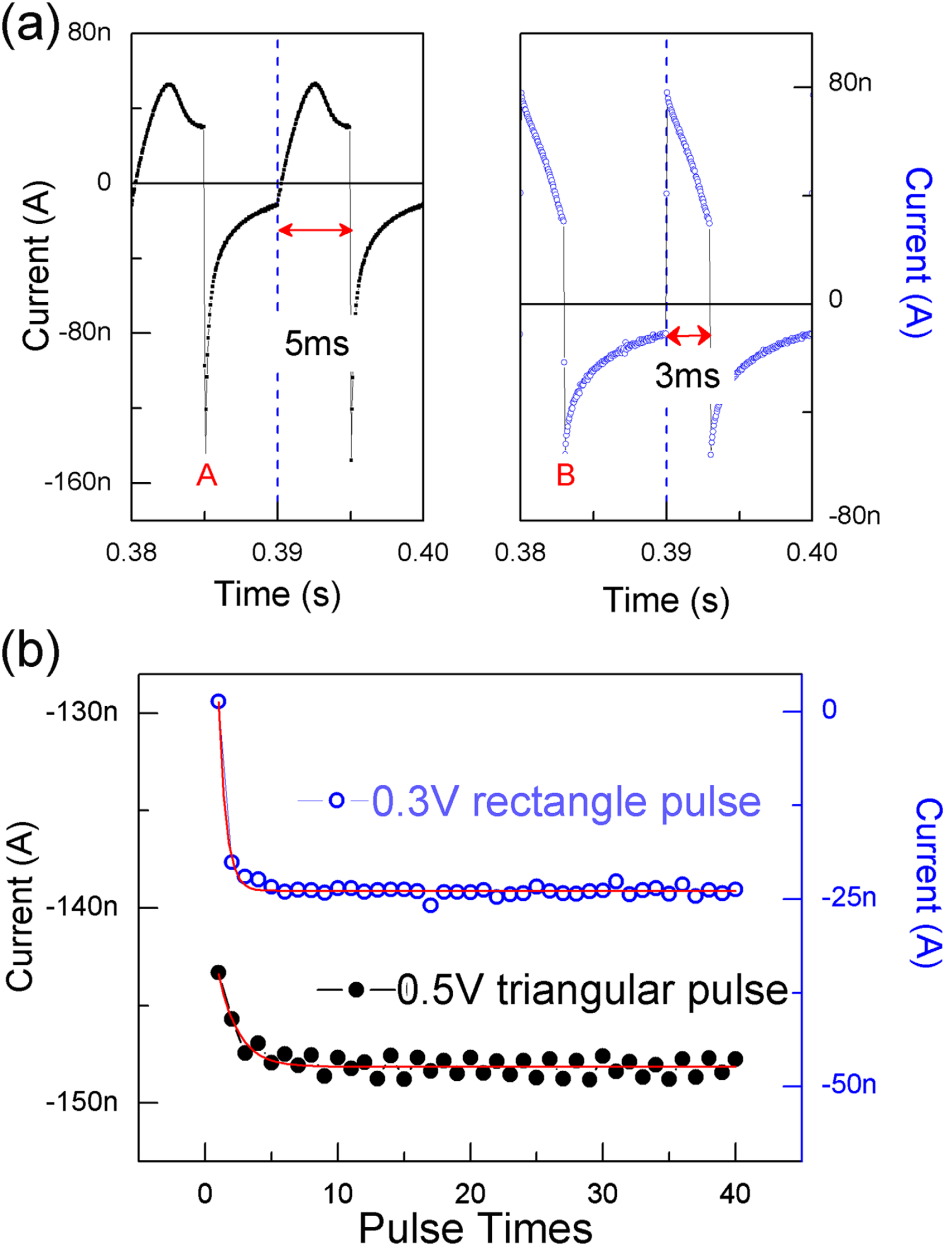
Pulse responses. (a) Responses to triangular pulses (black dots) and rectangular pulses (blue circles). The bias was increased from 0 to 0.5 V at a rate of 100 V/s and was immediately removed for the triangular pulse. The pulse width is 3 ms and the pulse height is 0.3 V for the rectangular pulse. Letters ‘A’ and ‘B’ indicate the discharging peaks after a pulse. (b) Variation in the discharging peaks with the pulse number. The frequency was 100 Hz.

DC measurements suggested that the hetero-junction cell was limited by ion mobility and could be modulated using input timing. Subsequently, we studied the pulse response using two types of pulses (0.5 V triangular pulses and 0.3 V rectangle pulse). Figure 3a shows that a single pulse response contained two parts: the current during the pulse width and the discharging current after the pulse. The latter was dominated by inversely migrating ions because neither electrons nor holes were injected by the external field. Therefore, the latter should have contributed directly to memory or learning because of ion migration. [25], [36] Consequently, we examined the weight change in the responses during various pulse stimulations. The ratio of a response to an arbitrary frequency or to a baseline frequency is the weight value. According to the weight calculating paradigm in neuroscience, [36], [37] the peak in the discharge process relates to both ions and transmitter dependent synaptic plasticity and learning. In neuroscience, the peak usually terminates as excitatory post-synaptic current (EPSC) or inhibitory post-synaptic current (IPSC). Therefore, to obtain significance for bio-learning, we simply used *I_A_* or *I_B_* in Figure 3a to calculate the weight modification without changing the input waveform. The response to a train of pulses with a 1 Hz frequency was used as a baseline (100% weight value). In addition, there was a technical reason for using this calculating paradigm. The two neighboring pulses did not influence each other for the response during baseline stimulations because the previous pulse response thoroughly decayed before the next pulse arrived. If the frequency were increased (higher than 1 Hz), the response from the later pulse began to overlap the response from the previous pulse and, subsequently, induced a gradual weight change. At this time, the weight calculation became significant.

Interestingly, when the pulse frequency was fixed, *I_A_* or *I_B_* was found to become stable with an increase in the pulse number. Either value increased with the pulse number to a limiting value, which could be fitted using an exponential decay curve (Figure 3b). This property differs greatly from results obtained in recent studies mimicking the frequency response using non-volatile memristors. In contrast, the weights calculated in those studies increased or decreased monotonically and quickly with the pulse number. [38]–[40] An external circuit, such as a compliance current circuit, is required to limit the increased weight for these cases. Without the protection of compliance current, the unlimited current would fuse the system when the stimulation number increased continuously.

**Figure 4:**
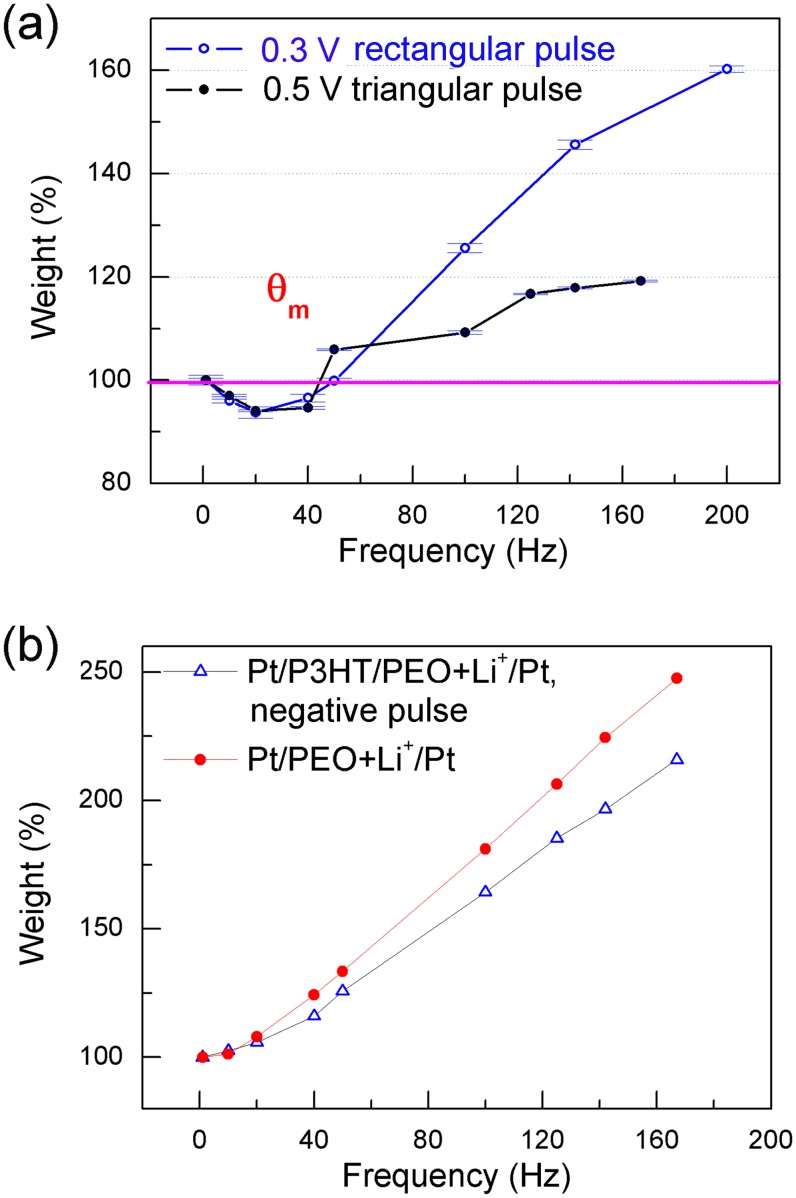
Weight variations dependent on pulse frequency. (a) Weight change calculated using triangular (black) and rectangular pulse (blue) described in Figure 3a. The value of *θ_m_* indicates the threshold from depression to potentiation. (b) Weight variations obtained from the responses during negative pulse stimulations for Pt/P3HT/PEO + Li^+^/Pt (blue triangles) and the positive pulse stimulations for Pt/PEO + Li^+^/Pt (red cycles).

Considering the frequency dependent response in Figure 3, we determined the weight changes of the responses and plotted them as a function of pulse frequency in Figure 4. A train of 40 pulses was used for each fixed frequency. The average of the last ten values of *I_A_* or *I_B_* in Figure 3a was used to calculate the weight values when the pulse sign was positive. The error bars were plotted for the weight values in Figure 4a. The weight was found to be depressed during low-frequency stimulations (LFS), in the range of 10 to 50 Hz, but the weight was potentiated during high-frequency stimulations (HFS) over 50 Hz as shown in Figure 4a. The threshold (*θ_m_*) at which the weight changed from depression to potentiation was approximately 50 Hz, depending on each individual device. Other calculated weight curves for different cells were provided for statistical reference in the Supporting Information (Figures S1 and S2). Therefore, the frequency selectivity was demonstrated in Figure 4a when the device was stimulated by positive pulses.

The reproducibility of this frequency selectivity and the degradation of the cell were also verified. The yield of the cells was about 80% for each batch. Distinct NDR phenomena appeared in all batches of cells, which indicates the frequency selectivity. There were 270 cells (out of 600 cells in all batches) that were used to conduct complete and continuous measurements from 1 to 160 Hz as shown in Figures 4, S1 and S2. The cells presenting frequency selectivity retained their properties in the ambient environment within two weeks.

When the pulse sign was negative, a variation resembling Figure 3b was also observed. The difference between this variation and the variation in Figure 3b was that the discharging current increased more quickly to a stable value after approximately three pulses. In Figure 4b, we did not average the last 10 weight values because the error bars were very small. Moreover, we were unable to obtain either the depression effect or weight reversal if we used the negative pulses, as shown in Figure 4b. A standard capacitor does not display properties resembling either Figure 4a or 4b. Therefore, the input-frequency-dependent response in Figure 4 should relate directly to ion migration, both in the PEO layer and at the semiconductor/electrolyte interface, and no frequency selectivity appeared when the device was stimulated by negative pulses.

More meaningfully, we have found that this selectivity was consistent with the results in Figure 1b, (i.e., the NDR effect only appeared under a positive bias in the measurement range). This property is also consistent with the feature that information flows only one way in a neural system. [25] The depressions observed during LFS under the threshold (*θ_m_*) were not reported in previous studies that mimicked the learning process using a single-stimulation procedure. [38]–[41] The depression effect was normally obtained by adding an inverse bias in these memristive systems. In experiments, a common feature of these systems is the lack of a reported NDR effect in the experiments. Because the calculated weights increased or decreased monotonically with stimulation number, it was not possible to naturally select a value as a baseline. This issue is normally addressed in a memristive system composed of a field effect transistor using the small current between the drain and source as a factitious baseline. [41] These systems have no selectivity and are not intrinsically adaptive; subsequently, these systems will lose stability in the absence of a complex external restriction.

**Figure 5:**
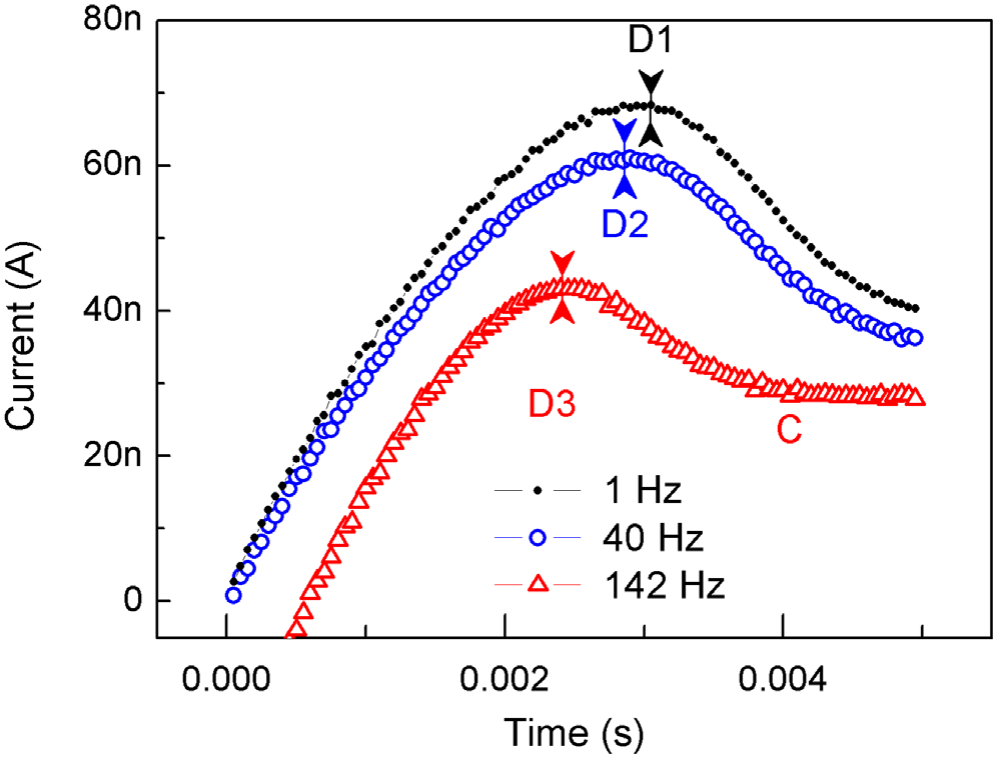
Current variations in the final pulse response. The triangular pulse responses were used with three typical frequencies, i.e., 1, 40 and 142 Hz, which correspond to the baseline, depression and potentiation states in Figure 4a, respectively. The start times of the last pulse responses were normalized to 0. The scope of Y axis is –5 nA to 80 nA.

We further proposed the mechanism governing the frequency selectivity in Figure 4a. If the DC inputs used in Figure 1 were replaced by a train of pulses, the ion migration was related to the two neighboring pulses. When the frequency was very low, (i.e., 1 Hz), the interval between two neighboring pulses was sufficiently long to allow the system to recover the initial electrolyte balance. When the frequency was increased, the two neighboring pulses coupled with each other. Generally, the second pulse arrived before the previous discharging was complete. Therefore, the previous discharging would overlap the subsequent input. If the charging and discharging processes between the two neighboring pulses arrived at equilibrium, their peak values approached a fixed value, and the response to the input frequency would be stable as shown in Figure 3b. This phenomenon is usually observed in the plasma system. The peak values in the charging processes (Figure 5) became lower when the frequency increased, which indicates that internal electric field E_i_ increasingly impeded the injection of electrons or holes, and ions accumulated more dramatically. Therefore, inverse ion migration dominated more effectively during the discharging process when the frequency increased. The maximum discharging strength after an input process (A or B in Figure 3a) should be determined by the accumulated ions and the inverse electric field at the P3HT/PEO + Li^+^ interface at the end of the previous input.

To understand the origin of the frequency selectivity in Figure 4a, we selected three typical input processes, which were abstracted from the pulse responses at the frequency of 1, 40 and 142 Hz in Figure 4a; for comparison, we plotted them in Figure 5. These frequency correspond to the baseline, depression and potentiation states, respectively. It is clear that the time of the charging process was brought forward when the frequency was increased. The timings for the charging peaks had the following relationship: *T_D3_<T_D2_<T_D1_*. The input curve for 142 Hz showed an inflexion of *T_C_* after *T_D3_*, which indicates that the discharging (or de-doping) in the NDR process (Figure 1b) had ended; consequently, the system was again driven to undergo a charging process (or re-doping) because of the stronger external field. The input curves for 1 and 40 Hz did not have such an inflexion, and the processes after *T_D2_* and *T_D1_* remained in the discharging (or de-doping) states. Because *T_D2_<T_D1_*, the discharging (or de-doping) period was longer in the input width when the pulse of 40 Hz was used, and more ions were repelled by the internal electric field. Consequently, we easily discerned the reason for depression with 40 Hz pulses and for potentiation with 142 Hz pulses (Figure 4a). Fewer ions had accumulated at the end of the previous input process for 40 Hz pulses than for 1 Hz pulses. However, the ions that accumulated for 142 Hz pulses were more numerous than for 1 Hz pulses. Therefore, the response to the triangular pulses in Figure 5 clearly exhibited the expected behaviors: the detailed charging and discharging processes and these processes’ relationship to the frequency selectivity. The timings of doping, de-doping and re-doping were modulated by the input frequency. Moreover, the response to rectangle pulses (Figure 3a) should include these doping, de-doping and re-doping processes in addition to their reciprocal coupling (modulated by the input frequency). However, these phenomena were masked by the rapid charging process (currents) at the beginning of the input process because of an abruptly strong electric field (Figure 3a). For this case, the rectangle pulse width should be carefully selected. If the rectangle pulse width were too longer, the effect of modulating the timings doping, de-doping and re-doping would be weakened, and then result in missing the observation of the depression under LFS.

The lack of frequency selectivity under negative pulses resulted because the NDR phenomenon did not appear in the measurement range. When a negative pulse was used, the CF_3_SO_3_^−^ could not be easily doped into P3HT unless a very large external voltage was applied. Consequently, the accumulation process of Li^+^ near the TE in our device was identical to the process in Pt/PEO + Li^+^/Pt sandwich. There was not ionic filter effect at the TE side. This case required a much higher external electric field to form a plasma sheath and, consequently, could not experience the input-frequency-modulated processes of doping, de-doping and re-doping.

That counter ions are repelled because of to heavy doping has been mentioned in previous studies. [32], [42] However, no study had ascertained that the input frequency could be used to this process. Moreover, the calculation paradigm we used approximated the paradigm in Ref. 37. Therefore, we clearly demonstrated that a simple redox process could be disintegrated to a series of individual events and applied to signal handling. Some experiments have attempted to greatly enhance the bias amplitude to obtain nonvolatile memory devices. [5] We hope to enhance polaron lifetime by doping a semiconducting polymer, (i.e., adjusting interfacial barrier or ion mobility without enhancing the bias values or the power consumption). Moreover, a ‘bi-directional’ device could be fabricated with the designation of Pt/PEO:Li/P3HT/PEO:X/Pt, in which X might be Li, K, etc. These efforts would lead us to the realization of a frequency-dependent learning protocol, such as spike-rate-dependent plasticity, with long-term effects [37], [43].

## Conclusions

In summary, we studied pulse responses of a P3HT/PEO + Li^+^ hetero-junction and revealed that dynamic doping in a semiconducting polymer could be modulated by input frequencies. The device response was stable when a train of pulses with a fixed frequency was applied. Frequency selectivity was observed firstly. The weight calculated based on the pulse responses was depressed during low-frequency stimulations, while the weight was potentiated during high-frequency stimulations. Therefore, the device responded adaptively and selectively to input signals. Detailed analysis of the pulse response showed that the input frequency could modulate the timing of ion doping, de-doping and re-doping at the polymer/electrolyte interface. We believed that the observed phenomena could be used for signal handling, or even for simulating synaptic plasticity and bio-learning.

## Supporting Information

Figure S1 **Weight calculated from pulse responses to triangular pulses with bias amplitude of 0.5 V.**
(DOCX)

Figure S2 **Weight calculated on the base of pulse responses to rectangular pulses with bias amplitude of 0.3 V.**
(DOCX)
