# Supplementary material for: Frequency Selectivity in Pulse Responses of Pt/Poly(3-Hexylthiophene-2,5-Diyl)/Polyethylene Oxide + Li+/Pt Hetero-Junction
Source: PLoS One. 2014 Sep 22;9(9):e108316. doi: 10.1371/journal.pone.0108316 (PMC4171527; doi:10.1371/journal.pone.0108316)
Supplement: Figure S1 — Weight calculated from pulse responses to triangular pulses with bias amplitude of 0.5 V. (DOCX) [file pone.0108316.s001.docx]

**Figure S1.** Weight calculated from pulse responses to triangular pulses with bias amplitude of 0.5 V

The pulse responses were measured by using various stimulation shapes. Figure S1 shows those by using triangular pulses with bias amplitude of 0.5 V. The weight value of 100 was obtained by using pulses responses to 1 Hz stimulations, and it was used as baseline.
