# Supplementary material for: Frequency Selectivity in Pulse Responses of Pt/Poly(3-Hexylthiophene-2,5-Diyl)/Polyethylene Oxide + Li+/Pt Hetero-Junction
Source: PLoS One. 2014 Sep 22;9(9):e108316. doi: 10.1371/journal.pone.0108316 (PMC4171527; doi:10.1371/journal.pone.0108316)
Supplement: Figure S2 — Weight calculated on the base of pulse responses to rectangular pulses with bias amplitude of 0.3 V. (DOCX) [file pone.0108316.s002.docx]

**Figure S2.** Weight calculated on the base of pulse responses to rectangular pulses with bias amplitude of 0.3 V.

Figure S2 show those by using rectangle pulses with bias amplitude of 0.3 V. The weight value of 100 was obtained by using pulses responses to 1 Hz stimulations, and it was used as baseline.
